# Effects of psilocybin therapy on personality structure

**DOI:** 10.1111/acps.12904

**Published:** 2018-06-19

**Authors:** D. Erritzoe, L. Roseman, M. M. Nour, K. MacLean, M. Kaelen, D. J. Nutt, R. L. Carhart‐Harris

**Affiliations:** ^1^ Centre for Neuropsychopharmacology Division of Brain Sciences Faculty of Medicine Imperial College London London UK; ^2^ South London and Maudsley NHS Foundation Trust London UK; ^3^ The Institute of Psychiatry, Psychology and Neuroscience Kings College London London UK; ^4^ Sherman CT USA

**Keywords:** depression, personality, NEO‐PI‐R, Openness, psilocybin, psychedelic

## Abstract

**Objective:**

To explore whether psilocybin with psychological support modulates personality parameters in patients suffering from treatment‐resistant depression (TRD).

**Method:**

Twenty patients with moderate or severe, unipolar, TRD received oral psilocybin (10 and 25 mg, one week apart) in a supportive setting. Personality was assessed at baseline and at 3‐month follow‐up using the Revised NEO Personality Inventory (NEO‐PI‐R), the subjective psilocybin experience with Altered State of Consciousness (ASC) scale, and depressive symptoms with QIDS‐SR16.

**Results:**

*Neuroticism* scores significantly decreased while *Extraversion* increased following psilocybin therapy. These changes were in the direction of the normative NEO‐PI‐R data and were both predicted, in an exploratory analysis, by the degree of *insightfulness* experienced during the psilocybin session. *Openness* scores also significantly increased following psilocybin, whereas *Conscientiousness* showed trend‐level increases, and *Agreeableness* did not change.

**Conclusion:**

Our observation of changes in personality measures after psilocybin therapy was mostly consistent with reports of personality change in relation to conventional antidepressant treatment, although the pronounced increases in *Extraversion* and *Openness* might constitute an effect more specific to psychedelic therapy. This needs further exploration in future controlled studies, as do the brain mechanisms of postpsychedelic personality change.

**Significant outcomes**

- Personality trait *Neuroticism* decreased, while traits *Extraversion*,* Conscientiousness* (trend‐level), and *Openness* all increased from baseline to the 3‐month follow‐up after psilocybin‐facilitated therapy for treatment‐resistant depression.
- An exploratory analysis revealed that the degree of *insightfulness* during the psychedelic experience predicted changes in *Neuroticism* and *Extraversion*.
- Where changes in *Neuroticism* and *Conscientiousness* are consistent with what has been observed previously among patients responding to antidepressant treatment, the pronounced increases in *Extraversion* and *Openness* might constitute an effect more specific to therapy with a psychedelic.

**Limitations**

- Relatively small sample size of 20 patients suffering treatment‐resistant depression.
- Open‐label design and absence of a control condition.
- Two‐thirds of the patients in this study were men, limiting extrapolation to the general population where rates of treatment‐resistant depression are marginally higher in women than in men.

## Introduction

Major depression is a commonly occurring disorder associated with high morbidity, socio‐economic burden, and rates of completed suicide 1, 2. It affects 10–15% of the general population 2, 3, 4 and has been ranked by The World Health Organization (WHO) as the fourth leading contributor to the global burden of disease 5, with a forecast of becoming number one by 2030 6. Almost half of the cost and disease burden caused by depression has been attributed to treatment‐resistant depression (TRD) 7, 8, typically defined as ‘a poor response to two adequate trials of different classes of antidepressants’ 9. TRD is associated with longer duration and higher severity of the disease, more protracted functional impairment, and poses a significant personal and public health problem 8. TRD affects about 30% of patients with major depression and up to 60% if TRD is defined as absence of remission 7, 10. The poor prognosis and socio‐economic burden associated with TRD give ground for research focusing on therapeutic interventions with alternative strategies to conventional pharmaceutical and therapeutic approaches.

Beginning in the 1990s 11, 12, neurobiological and psychiatric interest in classic serotonergic psychedelic compounds, such as psilocybin, N,N‐dimethyltryptamine (DMT), and lysergic acid diethylamide (LSD), gradually re‐emerged after decades of being suppressed 13, 14. Recent pilot studies point to the potential of psychedelic‐assisted therapy to treat conditions including tobacco 15 and alcohol 16 addiction, obsessive‐compulsive disorder 17, end of life anxiety/depression 18, 19, 20, major depression 21, and TRD 22, 23 – (see Carhart‐Harris & Goodwin, 2017 for a review 24). Intriguingly, the treatment effect in these trials appears to last for several months – much longer than the pharmacological presence of the actual compounds 25, 26.

Typically, psychedelic‐assisted therapy involves only one or two sessions in which a moderate to high dose of a psychedelic compound is given in a supportive environment 27, 28 with the intention of evoking ‘peak’ 29 or ‘mystical‐type’ 30, 31 experiences, characterized by disintegration of ego boundaries and an accompanying sense of connectedness 32, 33, oneness, or unity 34. This treatment paradigm differs from the approach of long‐term daily pharmacological intervention associated with conventional antidepressant medication.

The mechanisms underlying the long‐lasting therapeutic effects of psychedelic therapy remain unknown. There appears to be a relationship between the therapeutic outcome and the subjective experiences during the psychedelic sessions 18, 27, 35, 36, 37, 38. Moreover, psilocybin and LSD may increase the NEO‐PI‐R 39 personality trait *Openness to Experience* (or simply ‘*Openness’*) in healthy volunteers after a single dose 40, 41. Interestingly, and constituting a possible link between the quality of the experience and the impact on personality, in the subgroup of participants who had mystical experiences during their psilocybin session, *Openness* remained significantly higher than baseline more than 1 year after the session 40.

*Openness* is considered to be one of the five major dimensions of personality and is linked to *Openness* to new ideas and values, imagination, aesthetic appreciation, novelty‐seeking, non‐conformity, and creativity 39. In major depression, effective treatment with antidepressants has been shown not only to increase *Openness* scores but also to significantly affect three of the remaining four NEO‐PI‐R personality domains; decreasing *Neuroticism*, increasing *Extraversion* and *Conscientiousness*, with *Agreeableness* remaining unchanged 42.

The aim of the present study was to explore whether psilocybin with psychological support modulates personality parameters in patients suffering from treatment‐resistant depression, to investigate whether these changes relate to the quality of the psychedelic experience and to investigate whether such modulations could potentially help us understand the long‐lasting nature of psychedelic‐assisted therapy.

## Methods

The study was approved by the National Research Ethics Service (NRES) London – West London, sponsored and approved by Imperial College London's Joint Research and Complication Organisation (JRCO), adopted by the National Institute of Health Research (NIHR) Clinical Research Network (CRN), and reviewed and approved by Medicines and Healthcare products Regulatory Agency (MHRA).

### Participants

Twenty patients suffering treatment‐resistant depression were enrolled in this open‐label feasibility study. The main inclusion criteria were: unipolar major depression of at least moderate severity (17+ on the 21‐item HAM‐D) and no improvement despite two courses of pharmacologically distinct antidepressant medications for an adequate duration (6 weeks minimum) within the current episode. Main exclusion criteria were: a current or previously diagnosed psychotic disorder or an immediate family member with a diagnosed psychotic disorder. Five patients had previously tried a psychedelic drug; in four of five cases, this had occurred in recreational contexts in early adulthood. All but three had undergone psychological therapy/counseling 23.

### Study procedure

Full details of study procedures have been published previously 22, 23. Briefly, the first phase of screening involved a scripted telephone interview, which was used to prescreen for the major inclusion/exclusion criteria. Suited candidates were invited for a screening visit at the Imperial Clinical Research Facility (ICRF) at the Hammersmith hospital where informed consent was taken. A detailed history of both physical and mental (using MINI‐5) health, routine blood tests, ECG, urine test for drugs of abuse and pregnancy where relevant, breathalyzer, and a number of baseline assessments, including the NEO‐PI‐R, were acquired during this visit. Eligible patients attended a preparation visit, followed by two dosing sessions, separated by one week. In the first session, patients received 10 mg psilocybin, and in the second, 25 mg. The second session was the focus of the therapeutic process, as only this dose was predicted to induce lasting therapeutic effects. After capsule ingestion, patients laid with their eyes closed while listening to a music playlist 27. Two therapists adopted a non‐directive, supportive approach, allowing the patient to experience a mostly uninterrupted introspection. Patients came back one day and again one week after the 25 mg session for integration of the experience.

### Measures

Personality was assessed using the NEO‐PI‐R instrument 39, which covers 5 domains, *Neuroticism* (anxious, insecure, emotional), *Extraversion* (sociable, optimistic, talkative), *Openness to Experience* (or short: *Openness*) (curious, imaginative, creative), *Conscientiousness* (hard‐working, ambitious, persistent), and *Agreeableness* (good‐natured, cooperative, helpful). Each domain has six facets, each of which contains eight items that are rated by respondents using a 5‐point Likert‐type scale ranging from strongly disagree to strongly agree. NEO‐PI‐R raw scores were standardized as T‐scores (*M* = 50, SD = 10) using the combined‐sex norms reported in the NEO‐PI‐R manual. Cohen's d effect size was also calculated from raw scores: (Mean‐score_baseline_ − mean‐score_3‐months_)/((SD_baseline_)^2^ + (SD_3‐months_)^2^)^0.5^. The subjective experience under psilocybin was assessed using the altered state of consciousness questionnaire (ASC) 43. This self‐rated instrument captures the acute quality of the psychedelic experience and covers factors such as *insightfulness*,* blissfulness*, experience of unity, and spirituality. The 16‐item Quick Inventory of Depressive Symptoms, (QIDS‐SR16) was employed to assess depressive symptoms at baseline and at selected time points. Change in severity of depressive symptoms, as assessed with different measures, from baseline to follow‐up time points have been published separately 23.

### Analysis

Baseline vs. 3‐month follow‐up NEO‐PI‐R scores were compared using two‐tailed paired t‐tests, and false discovery rate (FDR) method with a threshold of 0.05 applied. Results are reported as mean ± SD. A standard threshold for defining treatment response (≥50% reduction in QIDS score from baseline) was used to separate patients into responders and non‐responders. Bivariate correlations were tested using Pearson's correlation coefficient; to test whether any of 4 ASC subfactors related to the ‘peak’ experience, *insightfulness*,* blissful state*, ‘experience of unity’, and *spiritual experience* 37 were related to changes in any of the NEO‐PI‐R trait scores (baseline vs. 3‐month follow‐up), and to test associations between changes in NEO‐PI‐R scores (trait and facet scores) and QIDS scores. These correlation analyses were uncorrected for multiple comparisons and should be regarded as explorative in nature.

## Results

Out of the 20 patients included in the trial, 18 met criteria for severe or very severe depression at baseline (QIDS‐16 score of ≥16), and the remaining two suffered ‘moderate’ depression (QIDS‐16 score ≥11, <16). As described in 23, one patient decided not to complete most follow‐up measures, including NEO‐PI‐R at 3‐month follow‐up, therefore leaving 19 complete datasets (6 females and 13 males; mean age = 44.7 ± 10.9; 27–64).

### Personality changes from baseline to 3‐month follow‐up

**Figure 1:**
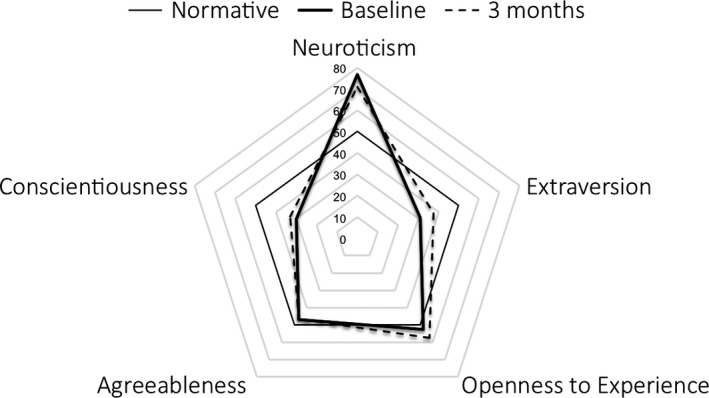
Trait T‐scores scores at baseline (solid thick line) and at 3‐month follow‐up (dotted line). T‐scores calculated with the use of means and standard deviations from a combined sample of 500 healthy women and 500 healthy men (NEO‐PI‐R manual). The normative data (solid thin line) are represented by normalized scores of 50.

**Figure 2:**
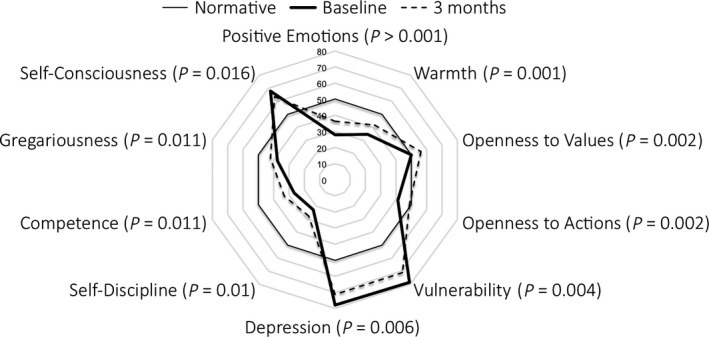
Facet T‐scores scores at baseline (solid thick line) and at 3‐month follow‐up (dotted line), with normative data normalised to 50 (solid think line).

As listed in Table 1 and illustrated in Fig. 1, three out of the five NEO‐PI‐R domain scores significantly changed from baseline to 3‐month postpsilocybin treatment: *Neuroticism* scores decreased (T‐score change: −5.7, *P* = 0.002), whereas both *Extraversion* and *Openness* increased (T‐score changes: 6.5, *P* < 0.001, and 4.9, *P* = 0.012, respectively). An increase in T‐score of 3.2 in *Conscientiousness* was only borderline significant (*P* = 0.086). Eleven NEO‐PI‐R facet scores, all arising from these four domains, changed significantly from baseline to 3‐month follow‐up, ten of these changes surviving FDR correction (these are listed in Table 1 and Fig. 2). Cohen's d effect sizes calculated from the raw scores are listed in Table 1.

**Table 1:** NEO‐PI‐R domain and facet scores pre‐ and post‐psilocybin

Domains and facets	Baseline T‐score	3 months T‐score	Change	*P*‐value	Cohen's *d*
Neuroticism (N)	76.6	70.9	−5.7	**0.002**	−0.571
Extraversion (E)	31.1	37.6	6.5	**0.000**	0.716
Openness to Experience (O)	52.7	57.6	4.9	**0.012**	0.437
Agreeableness (A)	46.8	46.7	−0.1	0.953	−0.006
Conscientiousness (C)	29.9	33.1	3.2	0.086	0.273
Anxiety (N1)	70.3	66.6	−3.7	0.037	−0.403
Angry‐Hostility (N2)	67.6	65.4	−2.2	0.222	−0.277
Depression (N3)	77.8	71.2	−6.6	**0.006**	−0.661
Self‐consciousness (N4)	68.1	63.6	−4.5	**0.016**	−0.366
Impulsiveness (N5)	57.8	56.3	−1.4	0.446	−0.184
Vulnerability (N6)	78.6	71.3	−7.3	**0.004**	−0.706
Warmth (E1)	34.7	41.8	7.1	**0.001**	0.604
Gregariousness (E2)	37.7	42.2	4.5	**0.011**	0.503
Assertiveness (E3)	43.9	45.9	2.0	0.152	0.304
Activity (E4)	35.2	38.3	3.1	0.071	0.442
Excitement seeking (E5)	43.1	44.9	1.8	0.279	0.171
Positive emotions (E6)	27.8	36.1	8.3	**0.000**	0.740
Openness to Fantasy (O1)	58.9	57.2	−1.7	0.415	−0.193
Openness to Aesthetics (O2)	53.2	56.2	3.0	0.157	0.244
Openness to Feelings (O3)	52.7	54.0	1.3	0.589	0.111
Openness to Actions (O4)	41.2	48.8	7.5	**0.002**	0.783
Openness to Ideas (O5)	51.5	55.2	3.7	0.077	0.324
Openness to Values (O6)	50.0	56.3	6.3	**0.001**	0.692
Trust (A1)	36.8	40.0	3.3	0.085	0.372
Straightforwardness (A2)	47.8	46.6	−1.2	0.478	−0.120
Altruism (A3)	44.8	43.2	−1.7	0.318	−0.177
Compliance (A4)	43.8	43.0	−0.8	0.685	−0.069
Modesty (A5)	58.3	55.9	−2.4	0.163	−0.199
Tender‐mindedness (A6)	56.2	58.9	2.7	0.274	0.215
Competence (C1)	26.7	33.0	6.3	**0.011**	0.743
Order (C2)	42.4	40.5	−1.9	0.376	−0.138
Dutifulness (C3)	38.2	41.3	3.1	0.191	0.250
Achievement striving (C4)	33.1	36.4	3.3	0.110	0.294
Self‐discipline (C5)	22.9	27.6	4.7	**0.010**	0.465
Deliberation (C6)	47.2	46.6	−0.6	0.662	−0.080

Domains and facets in bold represent scores that showed significant difference between baseline and 3‐month follow‐up with Student's paired *t*‐tests *and* that survived FDR correction for multiple comparisons. Thus, facet *anxiety* is the only of the 11 significantly changed facet scores not to survive the FDR correction. Cohen's *d* effect sizes were calculated from the raw mean and SD scores at baseline and 3‐month follow‐up.

### Personality changes vs. changes in depression scores

When dividing patients into clinical responders (*n* = 7) vs. non‐responders (*n* = 12) at 3 months, a group comparison revealed that among responders, *Neuroticism* score had decreased more from baseline to 3‐month follow‐up than among non‐responders (−23 ± 17 vs. −6 ± 8 respectively, *P* = 0.038), whereas *Conscientiousness* score had increased more among responders than among non‐responders over the same period (15 ± 11 vs. 0 ± 12 respectively, *P* = 0.017). Responders did not differ significantly from non‐responders with regard to personality changes within the 3 other domains (*Openness*: 11 ± 21 vs. 7 ± 6, *P* = 0.623; *Agreeableness*: −1 ± 7 vs. 1 ± 8, *P* = 0.577; *Extraversion*: 17 ± 10 vs. 9 ± 11, *P* = 0.105). In an exploratory analysis none of the 5 NEO‐PI‐R domain changes from baseline to 3‐month follow‐up significantly correlated with change in either QIDS or BDI depression scores at 3 months. There were positive associations between decreases in *Neuroticism* and decreases in QIDS scores (*r* = 0.41, *P* = 0.080), and a negative association between increases in *Extraversion* and decreases in QIDS (*r* = −0.42, *P* = 0.071). These relationships, however, did not reach statistical significance.

### Relationship between Peak Experience and personality changes

The degree of *insightfulness* experienced during the focal 25 mg psilocybin session was significantly associated with a reduction in *Neuroticism* score (*r* = −0.47, *P* = 0.043), as well as with an increase in *Extraversion* score (*r* = 0.54, *P* = 0.017). Also, *spiritual experience* was significantly positively correlated with increased *Extraversion* from baseline to 3‐month follow‐up (*r* = 0.47, *P* = 0.048), whereas positive relationships between *Extraversion* and scores of *blissful state* and *Experience of unity* only reached borderline significance levels (*r* = 0.41, *P* = 0.081, and *r* = 0.42, *P* = 0.075, respectively). None of the Peak Experience‐related ASC scores were associated with changes in *Openness* or *Conscientiousness*.

### Baseline personality scores as predictors of Peak Experience

Among the 5 NEO‐PI‐R domains assessed at baseline, only *Openness* showed a borderline significant association with any of the ratings of Peak Experience‐related ASC subdimensions, assessed in relation to the focal 25 mg psilocybin session; thus, a positive association between baseline *Openness* and *blissful state* reached borderline significance (*r* = 0.43, *P* = 0.063). In an exploratory analysis, we tested whether individual variation in *Openness* scores at baseline correlated with the Peak Experience. Correlation analysis revealed that two facets of this personality domain, *Openness to Fantasy* (*r* = 0.50, *P* = 0.030) and *Openness to Aesthetics* (trend‐level; *r* = 0.45, *P* = 0.054) were positively associated with *blissful state* experienced during the psychedelic experience. There were also trend‐level positive associations between *Openness to Fantasy* at baseline and both *Experience of unity* and *spiritual experience* during the experience (*r* = 0.41, *P* = 0.080, and *r* = 0.43, *P* = 0.069, respectively).

### Baseline personality scores as predictors of treatment response

*Neuroticism* scores at baseline showed a negative correlation with improvement in depression scores at 3 months at trend level (*r* = −0.42, *P* = 0.075), such that patients with higher *Neuroticism* scores at baseline showed reduced clinical improvement after psilocybin. Treatment responders after 3 months did not significantly differ from non‐responders on any of the 5 domain scores at baseline.

## Discussion

In this open‐label study of psilocybin therapy for treat‐resistant major depression, clinical improvement among patients was found to be accompanied by significant changes in personality measures. Thus, from baseline to 3‐month follow‐up, the NEO‐PI‐R ‘Big Five’ score of *Neuroticism* significantly decreased, *Extraversion* and *Openness* scores significantly increased, *Conscientiousness* showed a trend‐level increase, and no changes were seen in *Agreeableness*. A total of 11 of the 30 NEO_PI‐R facets also significantly changed, with 10 of these surviving multiple comparisons correction. To our knowledge, this is the first time personality measures have been reported to change among patients undergoing psychedelic therapy for depression. These results expand on the findings of psilocybin‐induced changes of personality traits in healthy volunteers 40.

Overall, the detected pre‐ to post‐treatment changes in both trait and facet scores in our trial corresponded well with observations from a study of patients who successfully underwent pharmacotherapy, mostly with selective serotonin reuptake inhibitors (SSRIs), for major depression 42. More specifically, the same four of ‘the Big Five’ traits changed in the two trials and in the same direction – that is toward the personality profile of healthy populations (although *Conscientiousness* only at trend‐level in our study). Also several of the facet changes we saw here overlapped with those reported by Costa et al. 42. More specifically, patients in both trials displayed decreases on the *Neuroticism* facets of *depression, vulnerability*,* self‐consciousness,* and *anxiety*. Increases in *Extraversion* included the facets of *warmth* and *positive emotions*. Increases in *Conscientiousness* included the facets of *competence* and *self‐discipline*. Means and standard deviations of NEO‐PI‐R scores were not reported by Costa et al., hindering calculation of effect sizes for direct comparison with our findings (listed in Table 1).

Pre‐ to post‐treatment changes in *Neuroticism*, a known vulnerability marker for affective disorders 44, 45, and increases in *Extraversion*, a trait associated with general positive affect 46, have previously been found to be significantly correlated with SSRI/SNRI‐induced reduction in depression severity 47. In accord with this, QIDS decreases in our study were associated with decreases in *Neuroticism* and increases in *Extraversion*, albeit at only trend‐level significance. In contrast, increased *Openness* did not correlate with treatment response and neither was it different between responders and non‐responders. This is consistent with the principle that *Openness to Experience* is orthogonal to anxiety or depression symptoms 46 – although there are also some findings to suggest there is a moderate relationship between *Openness* and psychological wellbeing 48, 49. Support for the change in Openness score potentially being an addition to, rather than a direct effect of, improved affective symptoms can be taken from a recent naturalistic survey among more than 200 individuals for whom symptomatology and personality measures were assessed online, of a psychedelic experience conducted by our team. Here, *Openness* changed equally for depressed and non‐depressed, whereas changes in Neuroticism were only observed among depressed individuals (manuscript in prep, data not shown).

In addition, an extensive meta‐analysis of personality changes after therapeutic interventions revealed that trait *Openness* was the only one among the ‘Big Five’ traits that did not robustly change 50. For a more direct comparison of our NEO‐PI‐R domain changes to previous treatment studies in depression, we identified 5 patient samples from 3 different trials in major depression where NEO‐PI‐R changes were reported after treatment with antidepressant medication 47, 51, 52 – although, in one of these studies, only *Neuroticism* scores were reported 51. Across these trials, SSRIs (citalopram, paroxetine, sertraline, and fluoxetine) 47, 51, 52, a norepinephrine‐dopamine reuptake inhibitor (bupropion) 51, 52, a serotonin–norepinephrine reuptake inhibitor (venlafaxine) 52, a reversible inhibitor of monoamine oxidase A (moclobemide) 51, a non‐selective irreversible monoamine oxidase inhibitor (phenelzine) 52, and a norepinephrine reuptake blocker (desipramine) 47 were used in relevant clinical doses over 8–20 weeks to treat a total number of 469 patients suffering major depression. The pre‐ and post‐treatment NEO‐PI‐R domain score data presented in these publications allowed for calculation of Cohen's d effect sizes.

Interestingly, changes in *Neuroticism, Agreeableness,* and *Conscientiousness* scores were very similar to what was seen in our study (sample size‐weighted average Cohen's *d*; −0.59, 0.04, and 0.18 respectively, vs. −0.57, −0.01, and 0.27 in our study). In contrast, Cohen's d effect sizes for *Openness* in our trial were more than 3 times larger (0.44 vs. an average of 0.13) and more than 2 times larger for *Extraversion* (0.72 vs. 0.32) than the average effect sizes from the comparison trials. Of note, the lack of full consistency regarding follow‐up (ranging from 8 to 20 weeks) means that interpretations of direct Cohen's *d* comparisons across studies should be made cautiously.

From the present data, it is hard to infer that the NEO‐PI‐R changes, and in particular changes in *Neuroticism* and *Extraversion, *represent trait and not state changes. However, a 3‐month enduring change might suggest that it might not just be a state artifact – a notion also supported by the long‐lasting psilocybin‐induced increases in *Openness* observed in healthy individuals 40.

Whereas the facet scores were not reported in these studies, none of the *Openness* facets were found to be significantly changed after successful antidepressant treatment in the study by Costa et al. 42. In contrast, the facets *Openness to actions* and to *values* significantly increased in our study. The facet *Openness to Actions* pertains to not being set in one's way, and instead, being ready to try and do new things. *Openness to Values* is about valuing permissiveness, open‐mindedness, and tolerance. These two facets therefore reflect an active approach on the part of the individual to try new ways of doing things and consider other peoples’ values and/or worldviews. Thus, treatment‐induced modification of *Openness* and its facets following psilocybin treatment might be an outcome separate and additional to the changes that have previously been seen with antidepressant treatment for example 50. Whether the pattern of change in personality measures, in particular with regard to *Openness*, is different between psychedelic therapy and conventional pharmacotherapy (e.g., with SSRIs) should be tested in the future. In our laboratory, we are currently setting up a treatment study in major depression where the effects of such treatment models can be compared directly.

Studies investigating the relationship between psychedelics and personality among non‐depressed individuals are worth considering in this regard. Follow‐up evaluations of work from the 1950–60s have suggested the possibility of long‐lasting effects on personality, resulting from the use of psychedelics 53, 54, and this has since been confirmed in a series of modern, controlled studies. For example, a single high dose of psilocybin with psychological support was found to facilitate long‐lasting increases in trait *Openness* in psychedelic‐naïve healthy individuals 40. Similarly, administration of LSD in a brain imaging setting led to discrete increases in *Openness* scores in healthy volunteers assessed at 2 weeks after the LSD session 41 although this was not replicated in a recent LSD study with longer postsession follow‐up time points (1 month and 12 months) 55. Recently, administration of the entactogen, 3,4‐methylenedioxy‐methamphetamine (MDMA), in conjunction with psychotherapy has been shown to lead to increases in *Openness*, as well as decreases in *Neuroticism*, in patients being treated for post‐traumatic stress disorder 56. Regular ceremonial use of the psychedelic brew, ayahuasca, was associated with higher *Openness* scores when compared with matched non‐ayahuasca using controls 57 and self‐reported lifetime recreational psychedelic use correlated positively with *Openness* scores in a large online survey 58, as well as in a separate brain imaging trial (D. Erritzoe, under review). Interestingly, in the latter trial, it was the *Openness to Actions* and *Openness to Values* subfactors that correlated with lifetime psychedelic use (D. Erritzoe, under review).

It is well established that trait *Openness* correlates reliably with liberal political perspective 58, 61, 62, 63. Given that psychedelics have been found to modulate *Openness* and other work has shown that *Openness* and liberal political perspectives are related, it is reasonable to surmise that psychedelics may modulate political perspective also. Indeed, work in the past has found associations between psychedelic use and attitudes of ‘personal liberty’ and ‘foreign policy liberalism’ 53, as well as concern for others, irrespective of culture of origin 62. Moreover, a series of recent studies have further endorsed a general relationship between psychedelic use and greater pro‐environmental behavior 63 and nature‐relatedness 58, 63, 64, as well as liberal 60 and antiauthoritarian perspectives 58, 64. The apparent link between *Openness* and a generally liberal worldview may be attributed to the notion that people who are more open to new experiences are also less personally constrained by convention and that this freedom of attitude extends into every aspect of a person's life, including their political orientation 65.

It is worth noting that the *Openness* score among patients entering our trial was already slightly higher at baseline (2.7 T‐score points higher, illustrated in Fig. 1) than the normative scores reported in the NEO‐PI‐R manual 66 and increased another 4.9 T‐score points following the intervention. *Openness* therefore differed from the other three personality traits in that it changed from an already higher than average baseline to an even higher level 3 months later. In contrast, the other traits all changed in the direction of normative data (see Fig. 1), for example, *Neuroticism* decreased from an abnormally high level at baseline, whereas *Conscientiousness* (trend‐level) and *Extraversion* both increased from an abnormally low baseline, and thus toward the values of healthy non‐depressed individuals 66. The explanation for *Openness* being relatively high at baseline may be found in the nature of the trial; people who are less *Open to Experience* are probably less likely to volunteer for a novel treatment involving a psychedelic drug. In support of this interpretation, healthy volunteers who took part in an invasive PET brain imaging study specifically had significantly higher *Openness* scores when compared to Danish NEO‐PI‐R norm data 67.

The observation that a single profound psychedelic experience can lead to lasting changes in personality is intriguing, especially when considering the relative stability of personality once adulthood is reached. Longitudinal studies have shown that personality changes after age 30 are typically subtle and/or gradual, that is around 1–2 T‐score points per decade, with a subtle drop in *Openness* and *Extraversion* scores and a slight increase *Agreeableness* in older age 68. However, as longitudinal studies of personality are typically conducted with very long intervals between sampling of personality traits, the personality literature overall provides limited evidence for how fast or slow personality trait change can occur 50. Thus, our observation of relatively marked changes in personality within a short time span challenges the assumption that personality can only change slowly, gradually, and subtly. A systematic review of more than 200 studies concluded that enduring and large changes in personality are obtainable through a range of therapeutic interventions 50 but to our knowledge, none so rapidly or as marked as with psychedelics. The phenomenon of psychological ‘quantum change’ may be relevant in this regard 69, where one's outlook and behavior rapidly and profoundly changes – such as with sudden religious conversion experiences.

Of the acute experience factors that most related to personality change, greater *insightfulness* during the 25 mg experience was found to be correlated with decreased *Neuroticism* as well as increased *Extraversion* at 3 months. Also, greater *spiritual experience* was correlated with increased *Extraversion*. Borderline relationships were seen between increased *Extraversion* and higher *blissful state* and *Experience of unity* scores. The observation that the long‐lasting impact of psychedelic therapy – in this case on measures of *Extraversion* and *Neuroticism* – may depend on their ability to occasion profound insights and ‘peak’ 29 or mystical‐type 70 experiences is supported by a number of modern clinical trials, where the magnitude of such peak or mystical experience is often predictive of positive clinical outcome 16, 18, 20, 35, 37. Problems related to the potentially non‐secular meaning of ‘mystical experience’ have recently been raised 24 but if the construct has predictive value, then it at least forces us to ask ‘why’? With the exception of *spiritual Experience*,* blissful state,* and *unity* predicting increased *Extroversion*, the major personality changes observed here were not strongly predicted by factors relevant to ‘mystical experience’. It is interesting that the somewhat more concrete notion of *insightfulness* showed more compelling relationships with the personality changes in the present study. Importantly, correlation analyses in this paper were *not* corrected for multiple comparisons and should therefore be considered exploratory in nature. In addition, it should here be noted that the sample size of the present study is modest, in particular when dividing it further into subgroups of responders and non‐responders. This limits our statistical power to detect small effect sizes that may be of clinical importance, and future randomized and placebo‐controlled trials with larger sample sizes will be important to further substantiate the findings from the present study.

These present findings are somewhat inconsistent with those of MacLean et al. who found that individuals who had a ‘mystical‐type’ experience were more likely to show sustained increases in *Openness* several months later 40. This may be explained by differences in the study populations, differences in the questionnaires that were used to assess subjective psilocybin experience, attitudes, and philosophy of support provided by the research staff (e.g., relative emphasis on mystical experience during preparation), or by the time delay at which personality was assessed (i.e., 3 months in the present study and over 12 in the MacLean study).

The neurobiological correlates of personality change after psychedelics have yet to be investigated. However, positron emission tomography (PET) imaging has revealed that brain serotonin 2A receptor levels, the key initiator of psychedelics’ signature psychological and neurophysiological effects 71 are positively associated with *Neuroticism* scores 67. In fact, the same *Neuroticism* facet scores that were found to decrease the most after psilocybin therapy in the present study (*vulnerability anxiety, depression,* and *self‐consciousness*) were also the facets most strongly correlated with serotonin 2A receptor binding in a sample of 83 healthy volunteers 67. Also, the trait ‘dysfunctional attitude’, which is associated with pessimistic beliefs, has been associated with elevated serotonin 2A receptor levels in both depressed patients 72 and individuals recovered from depression 73. It has been suggested that serotonin 2A receptor upregulation is secondary to deficient stimulation with serotonin 72, which is consistent with the observation that downregulation of 2A receptors is associated with treatment with antidepressant drugs that elevate synaptic serotonin (Muguruza et al., 2014).

To our knowledge, although *Openness* has not been directly associated with serotonin 2A receptor regulation, and 2A receptor levels did not appear to mediate an association between lifetime recreational use of psychedelics and elevated *Openness* scores (D. Erritzoe, J. Smith, P. M. Fisher, R. Carhart‐Harris, V. G. Frokjaer, G. M. Knudsen, under review), it is possible that serotonin 2A receptor function is linked to *Openness*. Evidence from rodents suggests that cognitive flexibility is in part mediated via the 2A receptor 74, and, as discussed previously, a variety of 2A receptor agonists, such as psilocybin, DMT, and LSD, are associated with increased *Openness*, as well as increased cognitive flexibility and creative thinking 75, 76, 77, 78, 79. Indeed, serotonin and more specifically, serotonin 2A receptor stimulation has been associated with a relaxing of prior assumptions 80 – which would fit with the qualities of open‐mindedness that characterize the *Openness* dimension. More investigations into the role of serotonin 2A receptors in mediating changes in *Openness* are therefore clearly warranted.

In summary, our study detected changes in personality measures from baseline to 3 months post psilocybin therapy in patients suffering major depression. Decreases in *Neuroticism* and (trend‐level) increases in *Conscientiousness* were consistent with what has been found previously among patients responding to antidepressant treatment, whereas pronounced increases in *Extraversion* and, in particular, in *Openness*, might constitute an effect more specific to therapy with a psychedelic than with other antidepressant interventions. This hypothesis needs to be explored further in future controlled studies, however, as do the brain mechanisms of postpsychedelic personality change. Finally, some preliminary evidence was found that certain changes in personality were predicted by the nature of the acute experience under psilocybin, with acute *Insight* being particularly implicated.

## Declaration of interests

None.
